# Genetic structure of Ponto‐Caspian trout populations shows gene flow among river drainages and supports resident *Salmo rizeensis* as a genetically distinct taxon

**DOI:** 10.1002/ece3.10335

**Published:** 2023-07-24

**Authors:** Levan Ninua, David Tarkhnishvili, Cort Lewis Anderson

**Affiliations:** ^1^ Institute of Ecology Ilia State University Tbilisi Georgia

**Keywords:** anadromy, brown trout, isolation by distance, Ponto‐Caspian region, resident forms, *Salmo caspius*, *Salmo labrax*, *Salmo rizeensis*

## Abstract

To assess the genetic structure of Ponto‐Caspian brown trout (*Salmo trutta* complex) populations, we analyzed both mitochondrial DNA sequences and genotypes at 10 microsatellite loci of fish caught in the Black Sea and from nine river catchments in Georgia, flowing into either the Black or Caspian seas. The results show that: (1) there is substantial genetic differentiation among Ponto‐Caspian trout populations, both among the populations of different nominal species and within those of the same species; (2) the genetic distance between conspecific populations from the Black and Caspian Sea basins exceeds that among the populations within the same basin. Moreover, within drainages, genetic distance correlates with the geographic distance; (3) the Black Sea itself is not a barrier to gene flow among the watersheds draining into the Black Sea; (4) some populations in the headwaters of the rivers draining into the Black Sea Basin fall out of this pattern and likely form a separate, non‐anadromous (resident) taxon, previously described from northeastern Turkey as *Salmo rizeensis*. This hypothesis is supported by mitochondrial DNA phylogeny. The presence of both anadromous and resident populations in a single river basin calls for a substantial re‐thinking of speciation patterns and taxonomy of Eurasian brown trout.

## Graphical abstract

The analysis of 238 samples of brown trout collected from 19 Georgian locations was studied by means of mitochondrial DNA sequencing and population genetic analysis based on the study of 10 microsatellite loci, showed intensive gene flow between drainages of the rivers flowing into the Black Sea, but not between the Black and Caspian Sea basins. The study identified a riverine form of brown trout, matrilineally monophyletic with *Salmo rizeensis* from NE Turkey and distinct from *Salmo labrax* dominated in the Black Sea basin.

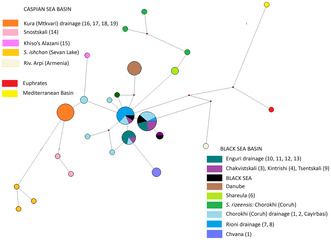

## 1 INTRODUCTION

The taxonomy of brown trout and its anadromous forms has been historically subject to much debate. Sabaneev (1875) used the names *Salmo salar*, *Salmo trutta*, and *Salmo fario* for marine, anadromous, and freshwater forms of brown trout. Subsequently, Black and Caspian Sea salmonids were recognized as anadromous forms of brown trout, originating from the drainages of these basins (Barach, 1962; Berg, 1959, 1962). Later, the name *S. salar* was retained for Atlantic salmon, the only distinct anadromous species which is absent from the Ponto‐Caspian region, where there live both anadromous and resident forms of brown trout designated as *S. trutta* (Bernatchez, 2001). *S. trutta fario* was the name used for the resident form of brown trout, irrespective of geographic origin (Jonsson & Jonsson, 2011).

In more recent decades, however, ichthyologists recognized a number of trout populations from Southern Europe, West Asia, and North Africa as separate species, distinct from the Central‐ and Northern European *S. trutta* (Kottelat & Freyhof, 2007; Segherloo et al., 2021; Turan et al., 2009). This number has increased to as many as 47 extant nominal species, and most of these taxa exhibit some level of genetic differentiation (Segherloo et al., 2021).

Within the Ponto‐Caspian area, 14 species of brown trout are formally described (Kottelat & Freyhof, 2007; Ninua et al., 2018; Segherloo et al., 2021; Turan et al., 2009, 2022), and two more taxa considered to be distinct, although non‐named species. However, validity of some of those, including *Salmo ciscaucasicus* from the Caspian Sea basin, as well as *Salmo coruhensis* and *Salmo rizeensis* from the Black Sea basin, was put under question (Segherloo et al., 2021; Table 1).

**TABLE 1:** Nominal species of trout from Ponto‐Caspian area, synonymy included.

Species	Description	Geography	Synonymy
*Salmo labrax*	Pallas, 1814	Black Sea basin	*S. fario*, *S. salar* (Sabaneev, 1875); *S. trutta labrax* (Berg, 1962); *S. trutta* (Bernatchez, 2001); *S. coruhensis* (Turan et al., 2020)
*Salmo coruhensis*	Turan et al., 2009	Black Sea basin	*S. fario*, *S. salar* (Sabaneev, 1875); *S. trutta labrax* (Berg, 1962); *S. trutta* (Bernatchez, 2001); *S. labrax* (Ninua et al., 2018; Segherloo et al., 2021, ^a^)
*Salmo rizeensis*	Turan et al., 2009	Black Sea basin	*S. fario* (Sabaneev, 1875); *S. trutta labrax* (Berg, 1962); *S. trutta* (Bernatchez, 2001); *S. labrax* (Segherloo et al., 2021, ^a^)
*Salmo* sp.	Segherloo et al., 2021	Danube basin	*S. fario* (Sabaneev, 1875); *S. trutta labrax* (Berg, 1962); *S. trutta* (Bernatchez, 2001)
*Salmo ezenami*	Berg, 1948	Northern Caucasus	—
*Salmo caspius*	Kessler, 1877	Caspian Sea basin	*S. fario*, *S. salar* (Sabaneev, 1875); *S. trutta caspius* (Berg, 1962)
*Salmo ciscaucasicus*	Dorofeeva, 1967	Caspian Sea basin	*S. fario*, *S. salar* (Sabaneev, 1875); *S. trutta caspius* (Berg, 1962); *S. caspius* (Segherloo et al., 2021, ^a^)
*Salmo* sp.	Segherloo et al., 2021	Caspian Sea basin	*S. fario*, *S. salar* (Sabaneev, 1875); *S. trutta caspius* (Berg, 1962)
*Salmo ischchan*	Kessler, 1877	Lake Sevan	—
*Salmo oxianus*	Kessler, 1874	Aral Sea basin	*S. trutta oxianus*, *S. trutta aralensis* (Berg, 1962)
*Salmo abanticus*	Tortonese, 1954	Lake Abant	*S. fario*, *S. salar* (Sabaneev, 1875); *S. trutta caspius* (Berg, 1962), *S. caspius* (Segherloo et al., 2021)
*Salmo araxensis*	Turan et al., 2022	Riv. Aras drainage	*S. fario*, *S. salar* (Sabaneev, 1875); *S. trutta caspius* (Berg, 1962), *S. caspius* (Segherloo et al., 2021, ^a^)
*Salmo murathani*	Turan et al., 2022	Riv. Aras drainage	
*Salmo ardahanensis*	Turan et al., 2022	Riv. Kura drainage	

^a^ In their paper, Segherloo et al. (2021) did not formally synonymized the species, but suggested conspecific status for *S. coruhensis*, *S. rizeensis* and *S. labrax*, as well as *S. caspius* and *S. ciscaucasicus*.

Ninua et al. (2018) investigated the differentiation of trout from rivers from both Black and Caspian Sea drainages, and the Black Sea coastal area of Georgia, including in their analysis published mitochondrial cytochrome *b* and control region DNA sequences of brown trout from the Ponto‐Caspian area. They synonymized *S. coruhensis* from the southern Black Sea drainage and *S. trutta* from Danube drainage with *Salmo labrax*, but did not question separate species' status for a non‐anadromous resident form, *S. rizeensis* (Turan et al., 2009). Ninua et al. (2018) also posit two species, *Salmo caspius,* and *S. ciscaucasicus*, in the rivers flowing into the Caspian Sea. Besides, they showed the monophyletic origin of all brown trout taxa from the Black, Caspian, and Aral Seas (Ponto‐Caspian basin), distinct from related fish from the Mediterranean, Persian Gulf, and Atlantic drainages. This interpretation was later supported by the genomic data of Segherloo et al. (2021).

The existing taxonomy reflects overall genetic differentiation among distinct geographic populations; however, it is unclear how the distribution of genetic variation and gene flow is partitioned among taxa, and whether differentiation among local trout populations scales with geographic distance, irrespective of formal taxonomy. Moreover, neither of the recent studies, except Turan et al. (2009), and the very old ecological observations of Barach (1962), addresses the genetic differentiation of anadromous versus resident populations. This is an important question: it is not entirely clear whether the anadromous life history is an inherent feature of certain populations of trout, or whether switching to anadromy is facultative. For *S. trutta*, existing analyses show very different results, suggesting either a facultative switch to anadromous life history, or population‐dependent life history (Jonsson & Jonsson, 1993), perhaps dependent on both, environmental conditions and certain genetic factors (Jonsson & Jonsson, 2006). For a related species, rainbow trout (*Oncorhynchus mykiss*) some genetic factors of anadromy have been identified (Hecht et al., 2013; Kendall et al., 2015; Le Bras et al., 2011; Nichols et al., 2008), giving rise to a complex polygenic inheritance. Earlier studies on *S. labrax* (Barach, 1962) suggest a facultative nature of anadromy in this species. By contrast, Turan et al. (2009), based on the analysis of mitochondrial DNA and phenotypic features, suggest the presence of both resident forms of the trout from the Chorokhi (Coruh) basin in the south‐eastern drainage of the Black Sea, coexisting with anadromous individuals.

We analyzed the population structure of trout from Georgia, sampling populations inhabiting the Black and Caspian Sea drainages, plus samples from the eastern coastal area of the Black Sea. These populations represent nominal *S. labrax*, *S. caspius* and *S. ciscaucasicus* (Ninua et al., 2018; Segherloo et al., 2021). To evaluate more precisely gene flow among the populations and the geographic distribution of genetic variation, mitochondrial DNA (cytochrome *b*) haplotypes were analyzed in tandem with 10 microsatellite markers. We addressed four working hypotheses: (1) the populations from the Black and Caspian Sea drainages are isolated. This hypothesis is supported based on prior mitochondrial DNA analysis (Ninua et al., 2018), but lacks evidence based on nuclear markers; (2) there is isolation by distance among populations from the same river drainage, reflected in the correlation between the genetic and geographic (riverine) distances; (3) the coastal area of the Black Sea is a barrier for migration from one river drainage to another; and (4) there are resident populations in the Black Sea basin, genetically distinct from the anadromous populations of the same drainage, that is, different species, as previously suggested by Turan et al. (2009).

## 2 MATERIALS AND METHODS

### 2.1 Sampling

**FIGURE 1:**
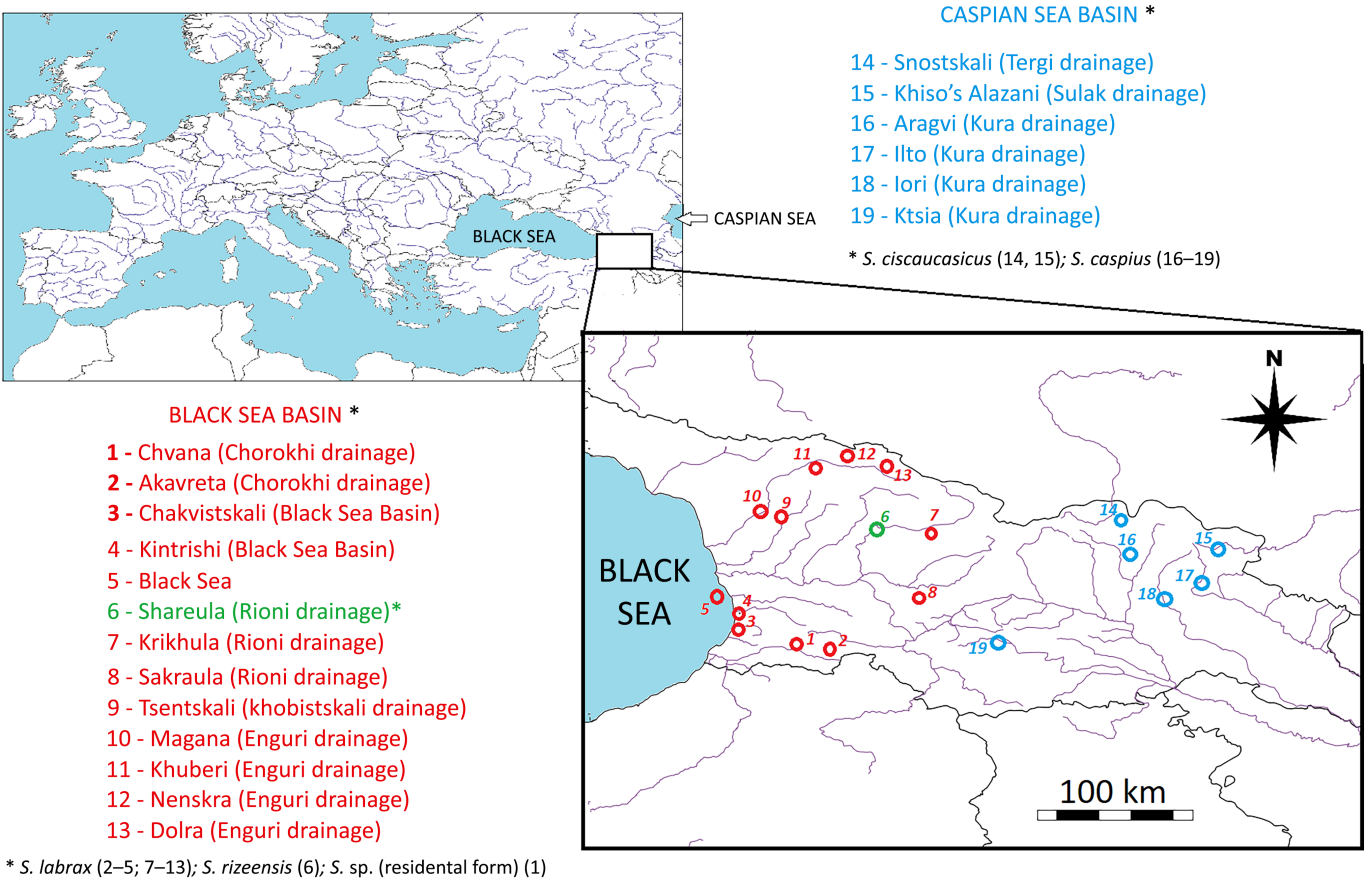
Sampling locations of brown trout: Colors show ultimate destination of drainage, red indicates Black Sea, blue indicates Caspian Sea. Population 6, shown in green, indicates the location of fish whose mitochondrial DNA clustered with *Salmo rizeensis* (Turan et al., 2009).

Fish were caught by electrofishing with a handheld portable device (EFGI 650: Bretschneider Spezialelektronik: http://www.electric‐fishing.de/index_e.html). Permits were obtained from the Ministry of Environmental Protection and Agriculture of Georgia (permit number 4288/01, effective April 30, 2019) and from the Agency of Protected Areas (permit number 876, effective August 17, 2018). We collected tissue samples of 238 individual fish from 13 locations in the Black Sea basin, and six locations from the Caspian Sea basin. The locations from the Black Sea basin were small rivers (1) Chvana and (2) Akavreta (riv. Chorokhi (Coruh) drainage); (3) river Chakvistskali; (4) river Kintrishi; (5) coastal area of the Black Sea; rivers (6) Shareula, (7) Krikhula, and (8) Sakraula (drainage of the riv. Rioni); (9) Tsentskali (drainage of the riv. Khobistskali); rivers (10) Magana, (11) Khuberi, (12) Nenskra, and (13) Dolra (drainage of the riv. Enguri). The locations from the Caspian Sea basin were (14) riv. Snostskali (drainage of the river Tergi (Terek)); (15) Khiso's Alazani (drainage of the river Sulak); (16) river Aragvi, (17) river Ilto, (18) river Iori, and (19) river Ktsia (drainage of the river Kura (Mtkvari)). From each location, 4–21 samples (12.5 on average) were collected (Figure 1). In addition, we analyzed 44 sequences of brown trout downloaded from GenBank (Table 2). Fish were fin‐clipped and then returned to their natural habitats. Fin clips were dried on filter paper and stored at room temperature until DNA extraction.

**TABLE 2:** The number of samples from each location and species used for the analysis of mitochondrial Cyt‐*b* gene and microsatellite genotypes (STR) at 10 loci.

Watercourse	Drainage	Location no.	Species	Cyt‐*b*	STR*
Our results					
Chvana	Chorokhi (Coruh) (BS)	1	*S. labrax* ^(?)^	3	10
Akavreta	Chorokhi (Coruh) (BS)	2	*S. labrax*	3 + 6^a^	21
Chakvistskali	Chakvistskali (BS)	3	*S. labrax*	3	15
Kintrishi	Kintrishi (BS)	4	*S. labrax*	10^a^	11
Black Sea	Black Sea	5	*S. labrax*	8^a^	14
Shareula	Rioni (BS)	6	*Salmo* sp. (*rizeensis*)	3	14
Krikhula	Rioni (BS)	7	*S. labrax*	3	11
Sakraula	Rioni (BS)	8	*S. labrax*	7^a^	9
Tsentskali	Khobistskali (BS)	9	*S. labrax*	3	14
Magana	Enguri (BS)	10	*S. labrax*	3	15
Khuberi	Enguri (BS)	11	*S. labrax*	4	6
Nenskra	Enguri (BS)	12	*S. labrax*	5^a^	7
Dolra	Enguri (BS)	13	*S. labrax*	3	15
Snostskali	Tergi (Terek) (CS)	14	*S. ciscaucasicus*	6^a^	17
Khiso's Alazani	Sulak (CS)	15	*S. caspius*	4	15
Aragvi	Kura (Mtkvari) (CS)	16	*S. caspius*	2^a^	12
Ilto	Kura (Mtkvari) (CS)	17	*S. caspius*	3	13
Iori	Kura (Mtkvari) (CS)	18	*S. caspius*	0	4
Ktsia	Kura (Mtkvari) (CS)	19	*S. caspius*	4^a^	15
Downloaded from GenBank					
Cayirbasi	Chorokhi (Coruh) (BS)	—	*S. coruhensis*	3	—
Kendirli	Iyidere (BS)	—	*S. coruhensis*	2	—
Sirli Stream	Euphrates (PG)	—	*Salmo* sp.	1	—
Ovit Stream	Iyidere (BS)	—	*S. rizeensis*	1	—
Cayeli	Cayeli (BS)	—	*S. rizeensis*	2	—
Rizekent Stream	Euphrates (PG)	—	*Salmo* sp.	1	—
Baltic Sea	Baltic Sea	—	*S. trutta*	3	—
Baltic Sea	Baltic Sea	—	*S. salar*	1	—
Lohnbach	Danube (BS)	—	*S. trutta*	2	—
Unknown	Iran (CS)	—	*S. caspius*	1	—
Göksu	Göksu (MS)	—	*S. trutta*	1	—
Caspian Sea	Caspian Sea	—	*S. caspius*	2	—
Arpa	Kura (Mtkvari) (CS)	—	*S. trutta*	1	—
Voidomatis	Aoös (MS)	—	*S. trutta*	1	—
Leksa	Leksa (AO)	—	*S. trutta*	1	—
Lake Ohrid	Lake Ohrid	—	*S. ohridanus*	1	—
Buna River	Buna River	—	*S. obtusirostris*	1	—
Soğuksu	Soğuksu (Md)	—	*S. platycephalus*	1	—
Zala	Zala (Ad)	—	*S. marmoratus*	1	—
N/A	India	—	*S. trutta*	5	—
North Sea	North Sea	—	*S. trutta*	1	—
Ims	Ims (AO)	—	*S. salar*	1	—
Dades	Dades (AO)	—	*S. trutta*	1	—
Tensift	Tensift (AO)	—	*S. trutta*	1	—
Oum er Rbia	Oum er Rbia (AO)	—	*S. trutta*	3	—
Isli Lake	Isli Lake (AO)	—	*S. trutta*	1	—
Moulouya	Moulouya (MS)	—	*S. trutta*	1	—
Ziz	Ziz (AO)	—	*S. trutta*	1	—
Ifni Lake	Ifni Lake (AO)	—	*S. akairos*	2	—

Abbreviations: AO, Atlantic Ocean; BS, Black Sea; CS, Caspian Sea; MS, Mediterranean Sea; PG, Persian Gulf.

^*^ Short tandem repeats.

^?^ Taxonomic status unclear.

^a^ Published in Ninua et al. (2018).

### 2.2 DNA extraction, PCR, sequencing, and microsatellite analysis

DNA was extracted from clipped adipose fins using the Qiagen DNeasy Blood & Tissue kit according to the manufacturer's instructions. Mitochondrial cytochrome *b* fragments of 30 fin samples were amplified using the following primers: nSsaL14437 (5′‐GCTAATGACGCACTAGTCG‐3′, Warheit & Bowman, 2008) and StrCBR (5′‐GGGGGCGAGRACTAGGAAGAT‐3′, Turan et al., 2009). Twenty microliter PCR reactions contained 3 μL of template DNA and primer concentrations 0.1 μmol/L. The details of PCR mix and amplification conditions are described in Ninua et al. (2018). The amplicons were sequenced in both directions on an ABI 3130*XL* automated sequencer using the same PCR primers and BigDye Terminator ver. 3.1. Sequences were aligned and edited with Geneious (ver. 8.1.9), and the unique sequences were deposited to GenBank (accessions # OP849668–OP849680). For phylogenetic analyses, an 841‐bp‐long region of cytochrome *b* was used. The sequences were obtained from 83 individuals collected in Georgia, and aligned with the 44 published homologous sequences of brown trout from western Eurasia, including those from the Ponto‐Caspian basin (Ninua et al., 2018; Turan et al., 2009); the total number of sequences from each drainage (river basin is presented in Table 2) with accession numbers in Appendix 1. Since Ninua et al. published 48 sequences from locations 1–19, the total number of sequences from the studied locations was 78.

For investigating population genetic structure, we amplified 10 microsatellite loci for 238 samples of trout collected from the sampled locations (Figure 1, Table 2). Each locus was amplified at least twice for each sample to ensure reproducibility. Microsatellite loci were amplified in three multiplex reactions using fluorescently labeled forward primers (Table 3). The 10‐μL final volume PCR mix contained 1 U of GoTaq Flexi DNA polymerase (Promega), 1× Colorless Flexi 5× buffer, and 2.5 mM final concentration of MgCl_2_. Amplification conditions were the same for all multiplex reactions: 3 min of initial denaturation at 95°C, followed by 30 cycles at 95°C for 20 s, 53°C with 0.2°C touchdown at each cycle for 30 s, and 72°C for 1 min. PCR products were mixed with formamide and LIZ 500 size standard (ThermoFisher). After 3 min denaturation at 95°C, samples were sequenced on ABI 3031*xl* genetic analyzer. For analysis, we used GeneMapper vers. 5.0 (ThermoFisher). We used the software MICRO‐CHECKER ver. 2.2.3 (Van Oosterhout et al., 2004) to check microsatellite data for large allele dropout and null alleles, separately for each of the study populations and each of the 10 studied loci.

**TABLE 3:** List of microsatellite locus names along with: repeat motif, multiplex group, primer melting temperature (*T*_m_), primer concentration (PC; μmol), fluorescent dye (Dye), primer sequences, and references.

Locus	Motif	Multiplex	*T*_m_	PC (μmol)	Dye	Sequences (5′–3′)	Reference
Ssa85	(CA)x(CG)x(CA)x	1	59	0.15	FAM	ACCCGCTCCTCACTTAATC	O'Reilly et al. (1996)
				0.15		AGGTGGGTCCTCCAAGCTAC	
Ssa408Uos	(GACA)x	1	56	0.15	VIC	CTCTTGTGCAGGTTCTTCATCTGT	Cairney et al. (2000)
				0.15		AATGGATTACGGGTACGTTAGA	
SsaD237	(TAGA)x	1	51	0.25	NED	CCTCTATCCATACAACACATGC	King et al. (2005)
				0.25		CAATGATGGAGTGGGAATTATC	
543AE	(CT)x	2	52	0.15	FAM	CTTTCTCTTGCGATAGTACGG	Estoup et al. (1993)
				0.15		GTTTCTACAGTCAGCACAAGTC	
SsoSL417	(GT)x	2	57	0.125	VIC	TTGTTCAGTGTATATGTGTCCCAT	Slettan et al. (1995)
				0.125		GATCTTCACTGCCACCTTATGACC	
SSsp2201	(GATA)x	2	60	0.125	VIC	TTTAGATGGTGGGATACTGGGAGGC	Paterson et al. (2004)
				0.125		CGGGAGCCCCATAACCCTACTAATAAC	
BG935388	(CAAT)x	2	54	0.125	NED	TGACCCCACCAAGTTTTTCT	Vasemaegi et al. (2005)
				0.125		GTTTAAACACAGTAAGCCCATCTATTG	
Ssa197	(GTGA)x(GT)x	3	57	0.2	FAM	TGGCAGGGATTTGACATAAC	O'Reilly et al. (1996)
				0.2		GGGTTGAGTAGGGAGGCTTG	
Ssa410Uos	(CAGA)x	3	54	0.25	FAM	CTACAATCTGGACTATCTTCTTCA	Cairney et al. (2000)
				0.25		GGAAAATAATCAATGCTGCTGGTT	
SSsp2216	(TAAC)x	3	60	0.2	NED	GCCAACAGCAGCATCTACACCCAG	Paterson et al. (2004)
				0.2		GGCCCAGACAGATAAACAAACACGC	

### 2.3 Phylogenetic analyses

We generated a mitochondrial phylogeny of the studied individuals with Bayesian inference (BI) using the software BEAST ver. 2.6.3 (Drummond et al., 2012). Settings for BEAST included: relaxed lognormal clock, Yule model, random distribution of the offspring number among the individuals; MCMC = 100,000,000; nucleotide substitution models were selected using MEGA software (Kumar et al., 2018). Additionally, we used the Median‐Joining approach (Bandelt et al., 1999) for networking individual haplotypes of the specimens, with software NETWORK 10.2.0 (Bandelt et al., 1999).

### 2.4 Population genetic analyses

Individual microsatellite profiles were subjected to principal component analysis (PCA), to visualize clustering of the populations in PCA space. The software used was SPSS 23.0 for Windows (IBM corp., 2015).

We used STRUCTURE ver. 2.3.4 (Pritchard et al., 2000) to evaluate the genetic structure across the Ponto‐Caspian populations. In the analyses we used an admixture model, assumed independent allele frequencies, and 50,000 iterations as burn‐in, with a total of 500,000 iterations. The number of potential population clusters (*K*) was set from 1 to 19 with five independent runs for each. The most probable *K* was chosen using the online tool Structure Harvester (Earl & VonHoldt, 2012).

The number of alleles, allelic richness, allele frequencies, and deviations from Hardy–Weinberg equilibrium (HWE) were evaluated using Arlequin ver. 3.5 (Excoffier & Lischer, 2010). The same program was applied to calculate the pairwise fixation index *F*_ST_ (1) between geographic populations of the Black Sea versus Caspian Sea basins and (2) among individual populations within each of the two sea basins, Caspian and Black, separately. In addition, we calculated the pairwise *R*_ST_ value (a modification of the fixation index for microsatellite loci; Gaggiotti et al., 1999; Slatkin, 1995) among the same populations. Sequential Bonferroni correction (Rice, 1989) was applied to the respective *p* values.

Finally, population genetic structure was evaluated by analysis of molecular variance (AMOVA) using ARLEQUIN ver. 3.5.2.2 (Excoffier & Lischer, 2010). We applied locus‐by‐locus analysis of genetic differentiation between the Black and Caspian Sea basins, among the populations from each of those basins, and among individuals of the same population (no individual level included), in order to infer whether the differences between the sea basins exceed the differences among the populations. We used 16,000 permutations for testing the significance of the obtained values; the distance matrices were computed based on different allele numbers (*F*_ST_‐like) and the sum of squared differences (*R*_ST_‐like), for validating the concurrence of the estimates based on these two metrics.

We used the models comparing two groups (Black and Caspian Sea basins) and six groups (population outliers from Chvana and Shareula, the rest of the populations from the Black Sea basin, populations from Kura, Tergi, and Sulak drainages).

### 2.5 Inferring isolation by distance

We used simple Mantel tests (Manly, 1986) to test whether isolation by distance best explains genetic structure of the sampled populations. We calculated correlation coefficients *R*_*xy*_ and the significance level *p* between two distance matrices: geographic riverine distances among the studied locations and genetic distances calculated as *F*_ST_ (among‐population component of departure of genotype frequencies from Hardy–Weinberg equilibrium not taking account of allelic differences) and *R*_ST_ (the departure while accounting for distances among alleles). Since populations from the of the Black and the Caspian basins appear to be effectively isolated, even if they are geographically close (Ninua et al., 2018), we separated analysis for the populations from rivers draining into these two seas. The riverine distances among the locations in the rivers independently flowing into the Black Sea (e.g., Chorokhi, Rioni, and Enguri) were calculated as the sum of the distances from the location to the respective river mouth, plus the distance along the Black Sea coast between the mouths of the two rivers. The same approach was used for the rivers drain into the Caspian Sea basin. To avoid possible nonlinear effects, prior to running Mantel tests, we log‐transformed the geographic distances. The software for running Mantel tests was zt (Bonnet & Van de Peer, 2002).

## 3 RESULTS

### 3.1 Mitochondrial DNA sequencing

**FIGURE 2:**
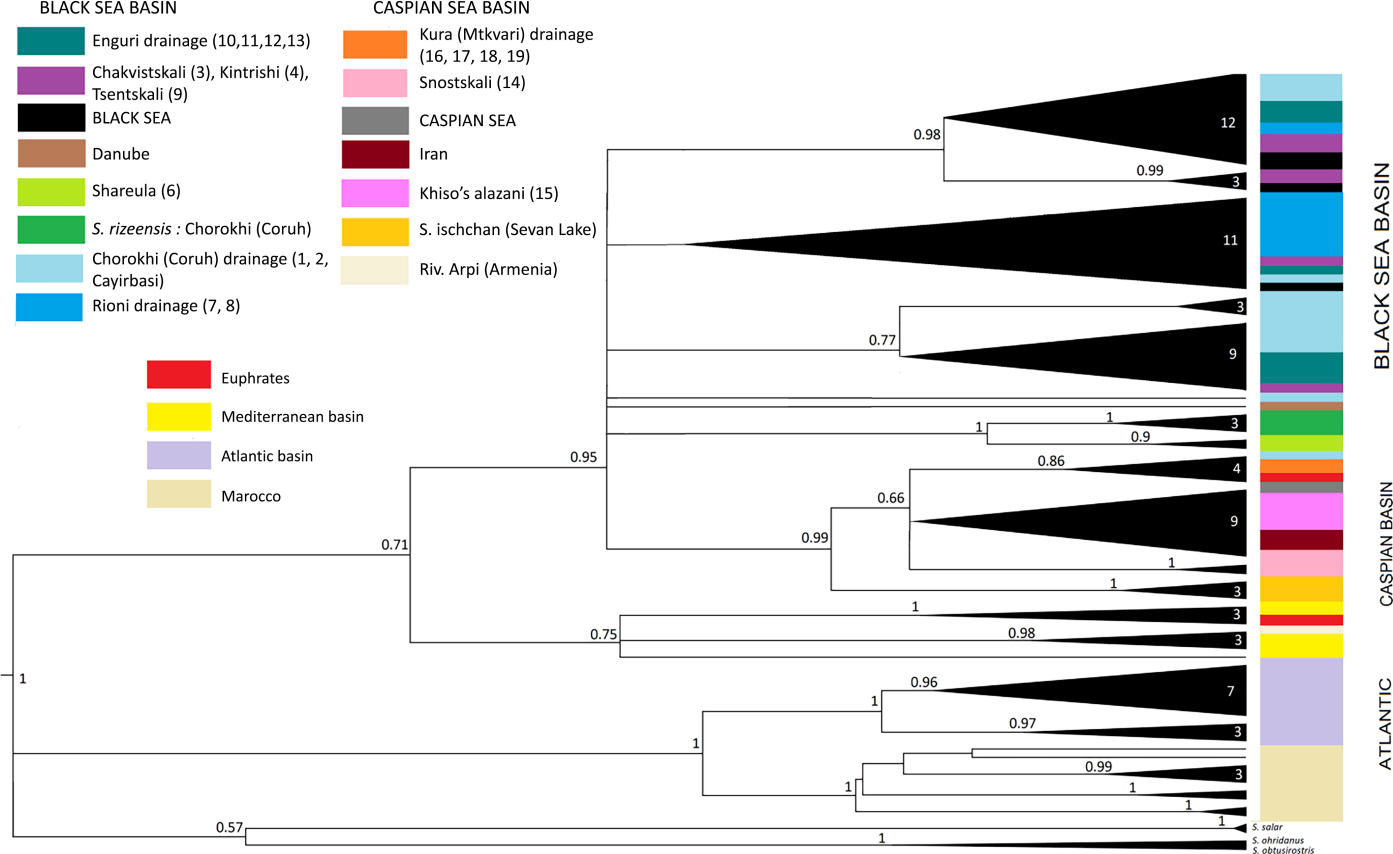
The Bayesian inference tree showing mitochondrial (cyt*b*) haplotype phylogeny in our samples and other published sequences of brown trout. The numbers on the triangles show the numbers of unique sequences from each cluster.

The Bayesian tree supported the monophyly of all trout from the Ponto‐Caspian basin (Figure 2). Within this clade, there were five well‐supported subclades: (1) fish from the Danube catchment; (2) fish from the drainages leading to the Caspian Sea; (3, 4) two clades containing most of the samples taken in the coastal area of the Black Sea and the rivers flowing into the Black Sea from the east (Chorokhi [Coruh in Turkey]; Rioni, Enguri, Kintrishi); one dominate with fish from Rioni River, another with fish from Chorokhi and few smaller rivers, and both clades have specimens caught in the Black Sea; (5) a clade containing nominal *S. rizeensis* sensu Turan et al. (2009), plus fish from the upper regions of the Rioni River (Shareula; Figure 1). In general, the data supported the conclusions of Ninua et al. (2018), suggesting the monophyletic origin of brown trout from the Ponto‐Caspian area, the monophyly of the fish from the Caspian Sea basin, and a high diversity of haplotypes from the Black Sea basin. Additionally, (1) the data clustered nominal *Salmo ischchan* with other fish from the Caspian basin; and (2) in contrast with the previous study, which did not discover the presence of haplotypes clustering with nominal *S. rizeensis* in Georgia, the current study identified a resident population from the upper region of the Rioni, which clustered with high statistical support with *S. rizeensis* from the Chorokhi headwaters in Turkey (Figure 2). Bayesian inference could not resolve (posterior probability < .5) the relative positions of the five described clades, suggesting a bush‐like pattern of evolution.

**FIGURE 3:**
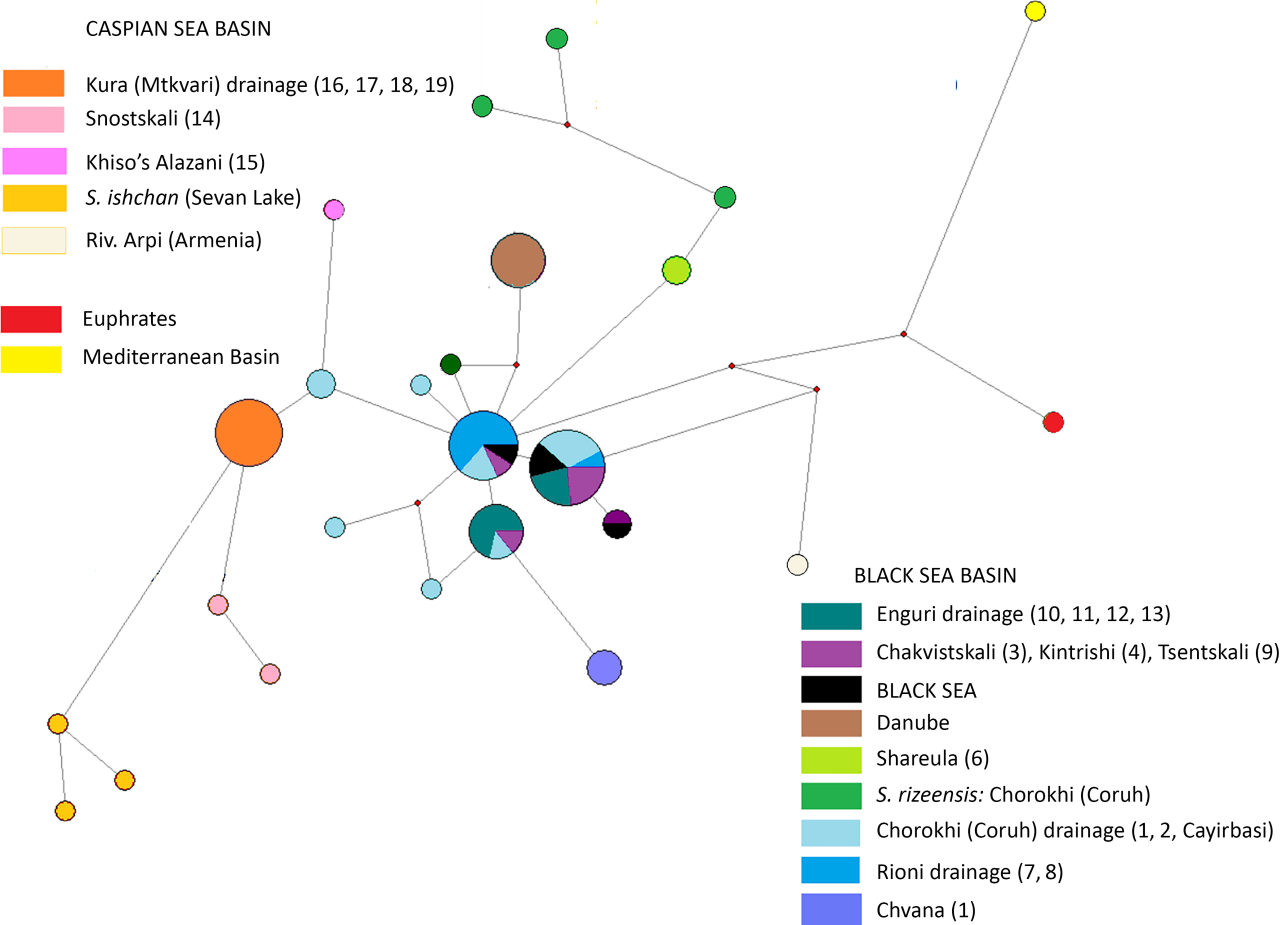
Median‐joining network linking the haplotypes of brown trout from the Caspian, Black Sea, and Mediterranean basins. The sampling locations of brown trout from the individual river basins marked with different colors (see the legend on the figure).

The haplotype network (Figure 3) depicts the situation more clearly. Haplotypes from the Caspian and Black Sea drainages are reciprocally distinct, with the exception of a single sequence from Armenia (Arpi) that is surprisingly close to the haplotype from the Mediterranean drainage. Within the Caspian Sea drainage, haplotypes from Snostskali (Figure 1; Tergi River basin, nominal *S. ciscaucasicus*) and that from Sevan Lake in Armenia (nominal *S. ischchan*) occupy a separate position, distinct from the haplotypes of Caspian Sea origin, Kura and Sulak River drainages, and from northern Iran samples, which share the same cytochrome *b* haplotype. The haplotypes from the Black Sea drainage are distributed among three most common haplogroups, shared among the specimens from the Black Sea and the main rivers (Chorokhi, Rioni, Enguri) flowing into it from the east, and nine rare haplotypes different from the “main” ones with one, two, or three mutated positions. The most deviated cluster of haplotypes from the Black Sea drainage is that marking nominal *S. rizeensis* from the Chorokhi drainage, and closely related to it, haplotypes from Shareula (Figures 1 and 3).

### 3.2 Microsatellite genotypes

There was no significant evidence for the presence of null alleles and large allele dropout for any population/locus. The number of alleles per locus within populations varied from 6 (for locus BG935388) to 34 (for locus SSsp2201). The mean allelic richness was the lowest (2.8) for the population from Chvana (Figure 1) and the highest (10.7) from the Black Sea (sample sizes 10 and 14, respectively). In the case of the populations from the Black Sea basin, the values of allelic richness were negatively correlated (Pearson *R*_*xy*_ = −.69, *p* ~ .02) with the riverine distance from the Black Sea coast. Genotype frequencies in none of the studied locations deviated significantly from Hardy–Weinberg equilibrium expectations.

**FIGURE 4:**
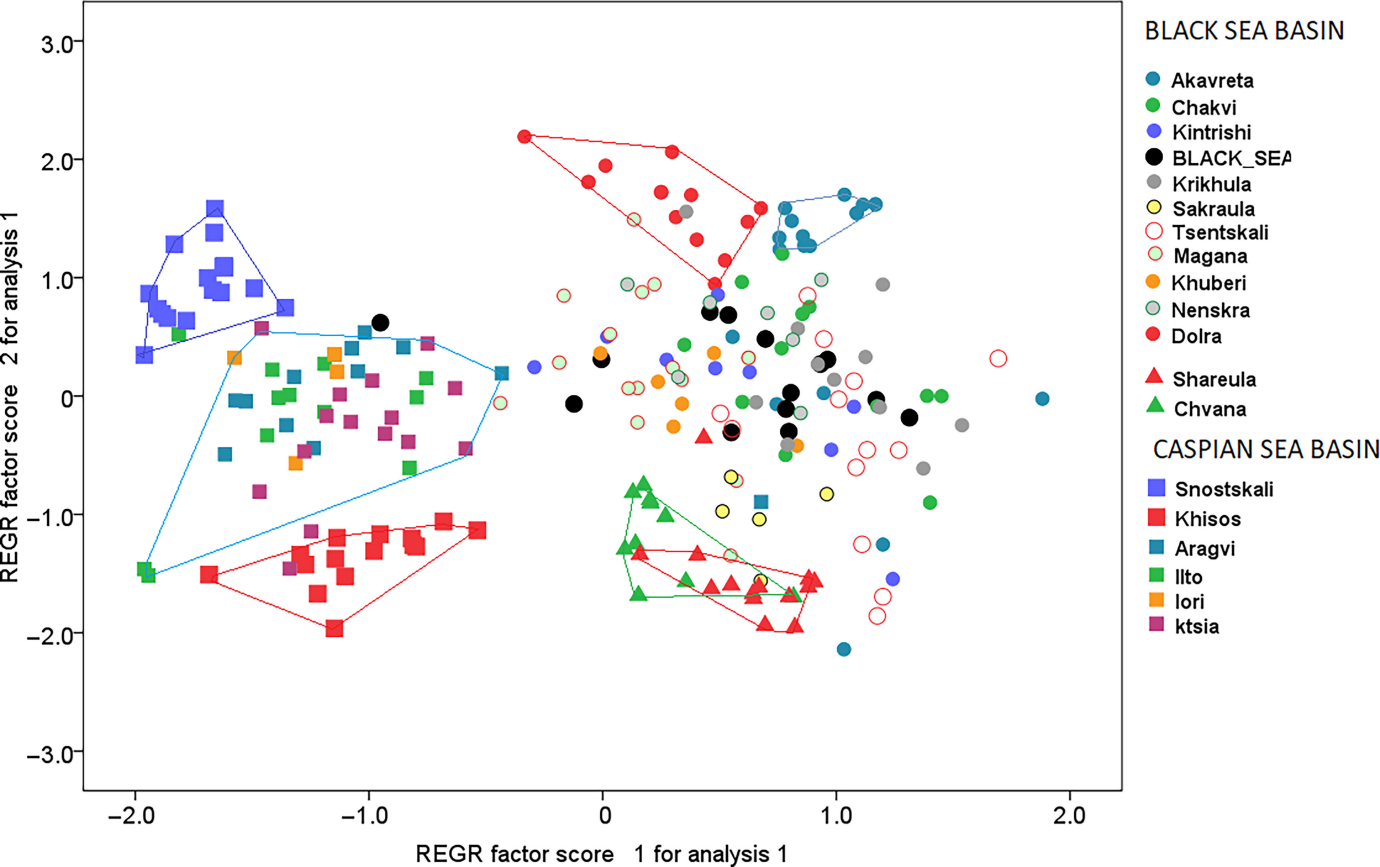
Ordination of the individual microsatellite genotypes along the first and the second principal component analysis axes. The first axis separates the individuals from the Black and the Caspian Sea basins, the second—individual populations within each of the two basins, including resident populations from Shareula and Chvana, as well as Akavreta and Dolra from the other populations of the Black Sea basin; and, populations from three river drainages independently flowing into the Caspian Sea.

Two first PCA axes explained 35.5% of the total variation in microsatellite alleles. Ordination along these axes showed (1) separation of the individuals from the Black and Caspian Sea basins along the first PCA axis, with the highest loadings on loci Ssa408Uos and BG935388; (2) the second axis, with the highest loading on the locus Ssa410Uos, separated genotypes of fish from the drainage of Tergi (location 14), Sulak (15) and Kura (16–19); and (3) the second axis separated genotypes from Shareula (location 6), Chvana (location 1), Akavreta (location 2), and Dolra (location 13) from the genotypes from other locations of the Black Sea basin (Figure 4). Whereas Akavreta and Dolra lay in the upper currents of the rivers Choroki and Enguri, Shareula and Chvana are closer to the Black Sea coast than the two former ones.

**FIGURE 5:**
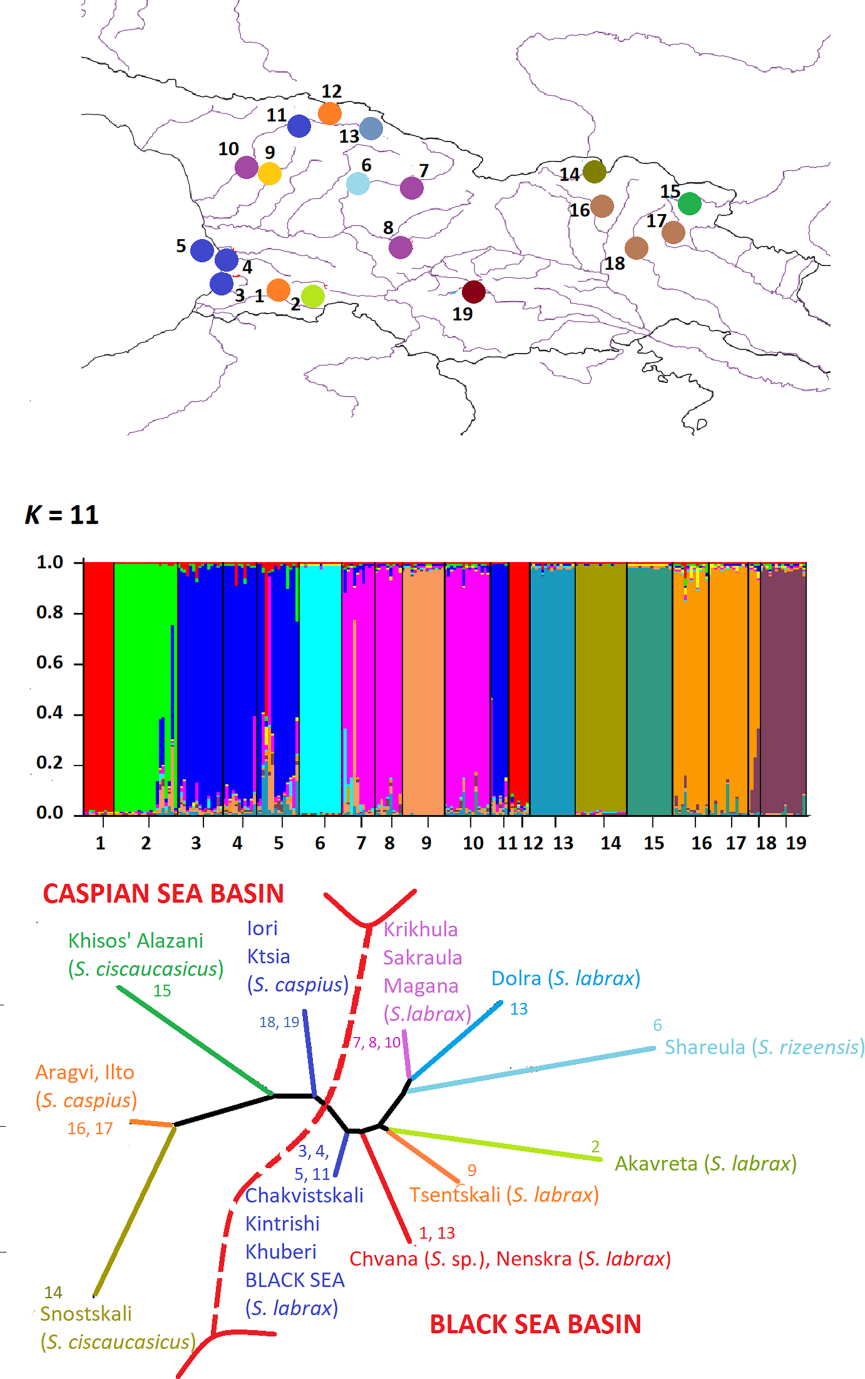
The allocation of the studied individuals to the 11 clusters (*K*) showing the highest delta log likelihood, inferred with STRUCTURE algorithm (considering 1 < *K* < 14). The non‐rooted tree reflects genetic distances among the inferred clusters (colored numbers) dominating in the locations marked with the black numbers. Colored dots on map are showing the origin of populations grouped in each cluster.

STRUCTURE analysis identified the highest delta log‐likelihood with *K* = 11 and the highest delta *K* with *K* = 13 (Appendix 2). In Figure 5 and Appendix 3, we present the assignment of individual fish to the inferred clusters with 1 < *K* < 13, in order to show the clusters that are separated at the lowest values of *K*. With *K* = 2, the populations are subdivided into those from the Black and the Caspian Sea drainages. With *K* = 5, the populations from Shareula and Chvana are allocated to separate clusters, and populations from the drainages of the Rivers Tergi and Sulak (Snostskali and Khiso's Alazani; Appendix 2) are separated from those from the Kura drainage. Remarkably, almost all individuals from all studied populations are almost fully assigned to one or another cluster, indicating the genetic distinctiveness of groups within the studied geographic area (Appendix 3). These separations remain until *K* = 11. With *K* = 11, the clusters identifying the populations from the Black and the Caspian Sea drainages are allocated in two distinct groups, and the cluster of location 6 is the most distant from the rest of the clusters representing Black Sea drainage (Figure 5).

**FIGURE 6:**
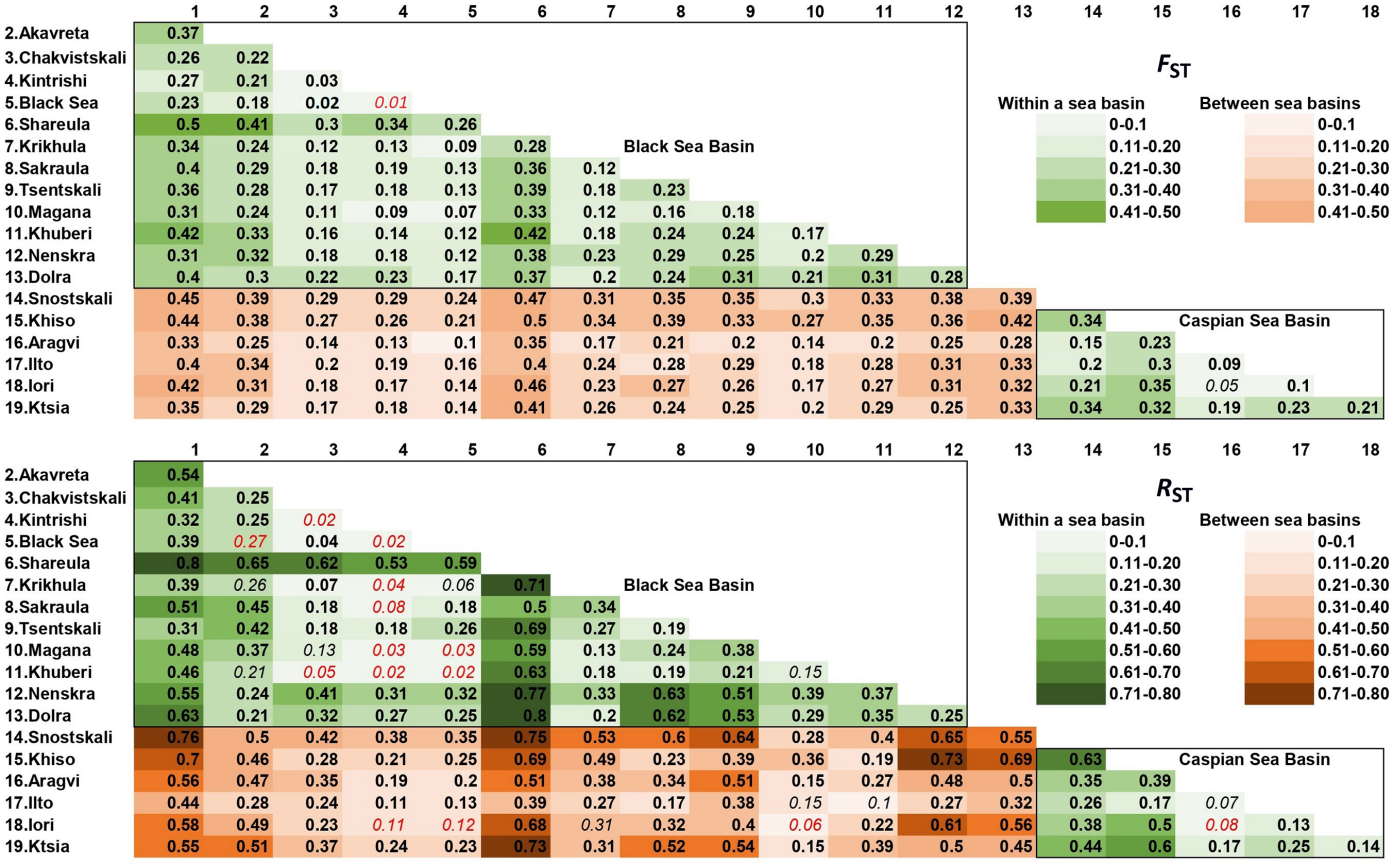
Pairwise measures of genetic differentiation for Ponto‐Caspian trout populations. Genetic distances within the same basin are shown with gradation of green and between sea basins with gradation of red. Upper panel—pairwise *F*_ST_ values; lower panel—pairwise *R*_ST_ values. For the most of the population pairs, *F*_ST_ *p* values < .01. Those where .01 < *p* < .05 shown in Italics. For insignificant values, red font is used.

Pairwise *F*_ST_ and *R*_ST_ values based on microsatellite genotypes are presented in Figure 6. Mean pairwise *F*_ST_ and *R*_ST_ values within the populations of the Black Sea basin were 0.236 and 0.300, respectively, while these values for the populations within the Caspian Sea drainage were 0.221 and 0.304, respectively. Average pairwise *F*_ST_ and *R*_ST_ values among the populations from the different sea basins were 0.286 and 0.395, respectively. The average values of the pairwise *F*_ST_ and *R*_ST_ among the populations from the different sea basins were significantly higher than these values calculated for the populations from the same sea basin (*t*‐test, *p* < .001). When the populations from Shareula and Chvana, which showed the highest genetic differentiation from the other populations of the Black Sea basin, were excluded from the Black Sea cluster, the pairwise *F*_ST_ and *R*_ST_ values dropped to 0.189 and 0.239, and when the populations of nominal *S. ciscaucasicus* were excluded from the Caspian Sea cluster, the pairwise *F*_ST_ and *R*_ST_ values dropped to 0.145 and 0.140. In most cases, the differences among the local populations are significant after stepwise Bonferroni correction is applied (*p* values < .05; Figure 6). Pairwise *F*_ST,_ was not significantly above zero only between Chakvistskali and Black Sea populations; pairwise *R*_ST_ values between populations from the Black Sea and from Chakvistskali, Krikhula, Kintrishi, Magana, and Khuberi varied between 0.02–0.06, suggesting close genetic relations between these rivers and Black Sea stock. Other insignificant values are shown in Figure 6.

**FIGURE 7:**
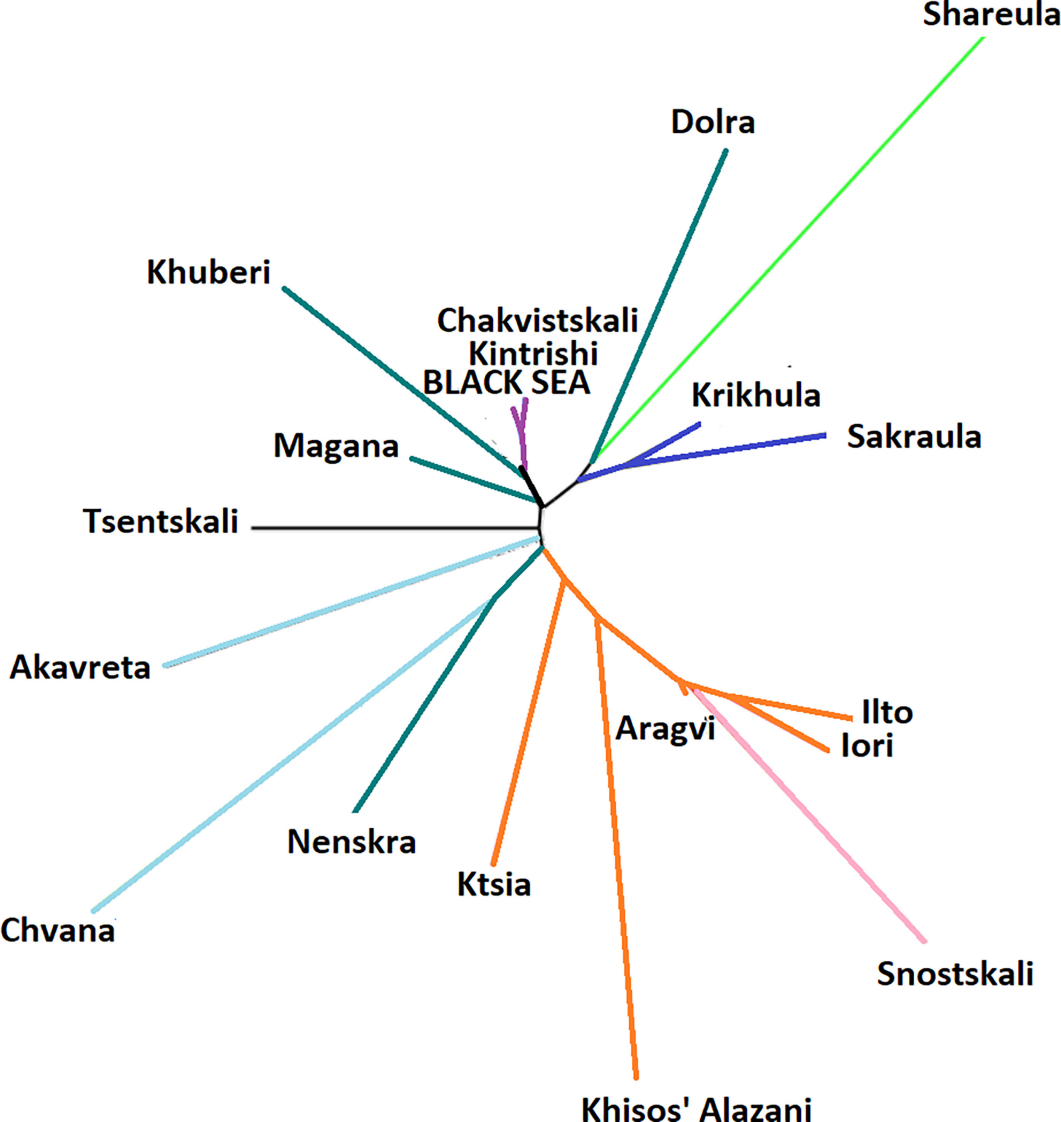
Neighbor‐joining unrooted tree based on pairwise *F*_ST_ of all sampled populations.

The unrooted neighbor‐joining tree based on the pairwise *F*_ST_ values among the studied populations is shown in Figure 7. The tree shows distinct clusters coinciding with the Black and Caspian Sea basins. However, the populations from individual river drainages flowing into the Black and Caspian Seas are not distinct. Some populations from Enguri, Rioni, and Chorokhi river drainages were genetically closer to the populations from the other and not the same river drainages.

The fish collected in the Black Sea were closest genetically to the populations from the small rivers entering the sea (#3 and #4 in Figure 1), and to one population from the Enguri drainage (Magana). The other finding of note is the high genetic distance of the population of Shareula from the rest of the Black Sea drainage populations, including those from the other tributaries of the same river (Krikhula and Sakraula). The average distance (*F*_ST_ estimates) between this population and the other populations from the Rioni Basin was 0.300, which substantially and significantly exceeded that for most of the other populations. Remarkably, fish from Shareula had a haplotype clustering with that of the nominal resident *S. rizeensis*. There was also another population (Chvana) quite distant from the other populations of the Black Sea drainage (average *F*_ST_ = 0.347).

AMOVA analysis results are shown in Table 4. The proportion of variance between Black and Caspian Sea basins showed 8% and 20% of the total variation (for *F*_ST_ and *R*_ST_, respectively) explained by the differences among the groups; this was lower than the variance between populations of the same group, especially in case of *F*_ST_‐based calculation (Table 4). We repeated the analysis after removal from the Black Sea basin population clusters populations from Shareula and Chvana, showing distinct genetic position from the populations of the same basins (as shown by Bayesian inference), pairwise *F*_ST_ calculation, and (in case of Shareula) clustered with mitochondrial haplogroup of *S. rizeensis*. In this case the component of the between‐basin variation *F*_CT_ increased from 0.077 to 0.083 (*F*_ST_) and from 0.209 to 0.252 (*R*_ST_).

**TABLE 4:** The output of AMOVA analysis based on the microsatellite profiles of the studied populations.

Source of variation	Sum of squares	Variance components	Percentage variation
Two‐group design, *F*_ST_			
Between the sea basins	107.469	0.357	7.708
Among locations within a basin	512.318	1.098	23.695
Within locations	1439.359	3.179	68.597
Average *F*‐statistics over all loci	Fixation indices		
Fixation indices	Significance (*p*)		
*F*_ST_: 0.31403	<.00001		
*F*_SC_: 0.25674	<.00001		
*F*_CT_: 0.07708	<.00001		
Two‐group design, *R*_ST_			
Between the sea basins	27,445.516	106.572	20.882
Among locations within a basin	82,411.491	189.252	37.083
Within locations	97,001.777	214.517	42.034
Average *F*‐statistics over all loci	Fixation indices		
Fixation indices	Significance (*p*)		
*F*_ST_: 0.57966	<.00001		
*F*_SC_: 0.46871	<.00001		
*F*_CT_: 0.20882	<.00001		

*Note*: The first design (two groups) compares trout from the Black and Caspian Sea basins. The second design (six groups) compares the outlier populations of the Black Sea basin (Shareula and Chvana), other populations from the Black Sea basin, drainages of the rivers Kura, Sulak, and Tergi (Caspian Sea basin).

### 3.3 Geographic isolation versus genetic distances

There was significant positive correlation between the ln‐transformed geographic (riverine) distances and pairwise *F*_ST_ values for the populations from the Black Sea drainage: (Mantel test, *R*_*xy*_ = .325, *p* = .007). For the Caspian Sea drainage, correlation was higher (Mantel test, *R*_*xy*_ = .510, *p* = .047). Correlation of *R*_ST_ values with geographic distance was lower (*R*_*xy*_ = .267, *p* = .008) than the correlation of *F*_ST_ with geography for the Black Sea drainage, but higher (*R*_*xy*_ = .599, *p* = .024) for the Caspian Sea drainage. Significant correlation between the geography and genetics for those locations that are not in the same river drainage means that the Black Sea is not an impenetrable barrier to migration among populations from different rivers. Moreover, the genetic distance between the fish found in the Black Sea or that from the locations of different rivers close to the sea was commonly smaller than the genetic distances among distant populations from within the same river basin. In other words, isolation by distance appears to be a stronger driver of genetic differentiation than water salinity/transit through the Black Sea.

## 4 DISCUSSION

Addressing the first hypothesis formulated in the introduction of this paper, we suggest that the populations of brown trout from the Black and the Caspian Sea basins are genetically isolated from one another. Previously, this hypothesis was confirmed based on mitochondrial DNA analysis (Ninua et al., 2018); additional mt‐DNA sequences obtained during this research also confirm this conclusion. Distinct position of these two groups reflected in PCA, the outcome of STRUCTURE clustering, and significant between‐group component of genetic variation, based on the microsatellite genotype analysis, additionally confirms with this hypothesis. This result is somewhat unexpected, since brown trout is a historically popular target for fishing and breeding (see Sabaneev, 1875), and has likely been subject to artificial relocation.

The analysis of microsatellite genotypes of the sampled populations also supports isolation by distance within different sea basins. Most of the brown trout populations are euryhaline (Berg, 1985), and sea water does not prevent gene flow among the populations from the different river catchments, independently flowing into the sea. This finding suggests the trout from both the Black and Caspian Sea basins can tolerate saline waters (although the salinity of both seas is much lower than that of the Mediterranean; Arkhipkin et al., 2013), and may at least occasionally switch to anadromous life history. Genetic distance among individual populations depends on the absolute riverine distance among the respective locations (including a coastal line of the Black or Caspian Sea), rather than on the geographic separation of the river catchments independently draining into a sea.

Finally, and most surprisingly, there are resident populations, distinct from the anadromous populations, consistent with the earlier suggestion of Turan et al. (2009), but contradicting the findings of Segherloo et al. (2021). Even more interesting is that the distinct resident and anadromous populations coexist in the same river catchment, without the presence of obvious geographic barriers between their locations. There are higher genetic distances between the presumably resident populations from the catchments of Chorokhi and Rioni (Chvana and Shareula) and other populations from the same catchments than among the anadromous populations from different catchments.

Segherloo et al. (2021) suggested the conspecific status of trout from the eastern part of the Black Sea drainage, synonymizing nominal *S. coruhensis*, *S. rizeensis* and *S. labrax* into a single species *S. labrax*. This is partly in accordance with findings of Ninua et al. (2018) who synonymized *S. labrax* and *S. coruhensis* but not *S. rizeensis*. Segherloo et al. (2021) proposed six other species from the Ponto‐Caspian area: *Salmo abanticus* from an isolated Abant Lake and *Salmo* sp. from Danube Basin (see also Schenekar et al., 2014), the fish from the rivers flowing into the Caspian Sea from the west and north (*S. caspius*), from the south (*Salmo* spp.), *S. ischchan* from Lake Sevan, and *Salmo oxianus* from the Aral Sea drainage. Our current results, based on the mitochondrial sequences, do not contradict these findings in the synonymization of *S. ciscaucasicus* and *S. caspius*. However, microsatellite profiles of the fish from the drainages of the rivers Tergi and Sulak are distinct related to the fish from a larger Kura drainage; besides, these forms are phenotypically more distinct than some species recognized by Segherloo et al.—see Ninua et al. (2018) for details. Our results confirm synonymization of *S. coruhensis* with *S. labrax*. However the species status of *S. rizeensis*, which Turan et al. (2009) described as a resident species, reflects real genetic differentiation; *S. rizeensis* shares a mitochondrial haplogroup with trout from the disjunct location Shareula (Rioni River drainage; Figure 1), which genetic distance from other populations of the Black Sea basin, based on the analysis of microsatellite multilocus genotypes, exceeds the genetic distance between the trout from the Black and Caspian Sea basins. We suggest that trout from this location, along with resident fish from northeastern Turkey, belong to a distinct species, *S. rizeensis*.

Elliot (1994) showed that resident and anadromous forms of brown trout coexist (and, probably interbreed) in many rivers draining into the Atlantic. Jonsson and Jonsson (2006) suggest that the resident versus anadromous life history may depend on the presence of physical barriers separating river habitat from the sea, or simply on the distance from the particular location to marine habitat; they also can co‐occur in the same river. The resident and anadromous morphs differ in adult mass (Jonsson, 1985); age at maturity (Jonsson & Jonsson, 2006); and egg size (Olofsson & Mosegaard, 1999); Jensen et al. (2019) suggested that trade‐off between fish mortality and reproductive success is a trigger of switching between the life histories. Jonsson and Jonsson (2006) also suggest the presence of genetic differences between the resident and anadromous morphs based on some differences in heritable characters, although environmental conditions can also lead to switching between the life histories. In particular, water level in a river may trigger change to and from anadromy (Jonsson et al., 2018). Findings of King et al. (2021) showed that commercially bred brown trout, stocked into a natural habitat of anadromous *S. trutta*, remain genetically distinct for at least 13 years after the sticking. This indirectly supports the presence of genetic differences between the non‐migratory and anadromous populations of trout. The other species of the genus *Salmo*, Atlantic salmon (*S. salar*), is usually anadromous and rarely produces resident forms; however, resident forms of *S. salar* are landlocked and isolated from the anadromous fish, most likely, since early Holocene (Berg, 1985). There is evidence for different gene pool in resident and anadromous forms of *S. salar*, including a higher allelic richness in populations of the latter form and stronger genetic differences between the resident populations (Ozerov et al., 2012; Tonteri et al., 2007).

The genetic basis of anadromy is better studied in rainbow trout (*O. mykiss*) from the Pacific Ocean; in this species, multiple genes are identified that influence life history; anadromy can be present or absent within the same polymorphic populations, depending upon unique constellations of alleles aggregating in individuals (Hecht et al., 2013; Le Bras et al., 2011; Nichols et al., 2008). Arostegui et al. (2019) showed that steelhead (the anadromous form of *O. mykiss*) is ancestral form related to the residential rainbow trout, and transition to the resident freshwater life history is secondary rather than primary in this group.

To conclude, common and independent change between the anadromous and resident life histories in trout is a multifactorial process that may occur both between conspecific populations, and also individuals of the same population, and is determined simultaneously by genetic and environmental factors.

Much less is known about the variation in life history and reproductive strategies of brown trout from the Ponto‐Caspian drainages, including wide‐spread *S. labrax* and *S. caspius*, than in the *S. trutta* from the Atlantic drainage. Sabaneev (1875) considered Black Sea salmon and brown trout from the Black Sea drainage as two different species, and only in the 20th century it was shown that they are two forms of the same species (Barach, 1962; Berg, 1959). Turan et al. (2009) was the first author who adduced two genetically distinct groups of fish in the south‐eastern drainage of the Black Sea: anadromous *S. coruhensis* (synonymized later with *S. labrax*) and resident *S. rizeensis*, marked with a distinct mitochondrial haplogroup. Ninua et al. (2018) showed that this haplogroup is not less distant from the other haplogroups of the Black Sea trout (nominal *S. labrax*) than reciprocally monophyletic brown trout haplogroups from the Black and the Caspian/Aral Sea drainages. These observations and the analysis of recombinant alleles reported in this paper support the maintenance of genetic differences between resident and anadromous trout.

The present study identified two populations, in the drainages of the geographically distant Chorokhi and Rioni (Chvana and Shareula), showing high genetic distances, both from each other and from other trout populations from the Black Sea drainage. The pairwise *F*_ST_ of each of these populations to the Black Sea population is 0.23–0.26, whereas the distance of the other 10 river populations to the Black Sea population varies between 0.01 and 0.18, depending on the distance from the sea coast. These distances are comparable with the genetic distance between *S. labrax* and *S. caspius*. One of these populations (Shareula) is marked with a mitochondrial haplotype that clusters with *S. rizeensis* (Turan et al., 2009).

These facts drive the conclusion that Shareula from the Rioni drainage (and, perhaps, Chvana from the Chorokhi drainage) are effectively isolated from the rest of the populations from the Black Sea basin, and are probably genetically determined residential forms similar to that described as *S. rizeensis* by Turan et al. (2009). Considering multiple evidence of spontaneous switching between anadromous and residential life histories in Atlantic brown trout (*S. trutta*) and the differences between rainbow trout and steelhead (*O. mykiss*), the presence of a similar pattern in *S. labrax* is not surprising. Haplotype divergence between *S. rizeensis* and “*S. coruhensis*” described in the paper of Turan et al. (2009) suggests that the divergence between the forms with different life histories occurred in the early to middle Pleistocene (Ninua et al., 2018). Another challenge is the sister status of *S. rizeensis* and our population from Shareula, based on mitochondrial DNA sequencing. This indicates that the divergence between the nominal *S. rizeensis* and *S. labrax* may precede their isolation as a result of their different life histories. Haplogroups from Chorokhi and Rioni River drainages, marking the *S. rizeensis* lineage, likely represent a monophyletic taxon that once could migrate from river to river through the coastal area of the Black Sea. Considering that during the glacial periods, the salinity of the Black Sea was substantially lower than now (Soulet et al., 2010) this could well happen. The questions remain—what was the trigger of separation between the two lineages, both of which could migrate through the coastal zone; and why might one of these lineages, in different parts of its range, turn from anadromous to residential life history. The reasons for the lineage separation within Ponto‐Caspian trout are discussed in Ninua et al. (2018). In fact, there are at least three almost equidistant mitochondrial lineages in the Black Sea trout, including *S. rizeensis*, and those lineages could emerge in case of temporary impenetrability of the Black Sea area, perhaps as a result of ice covering a large part of its surface during the periods of glaciation (Briceag et al., 2019) or alternatively during interglacials when Black Sea water was brackish and oxygen deficient due to impact of Mediterranean water inflow (Hoyle et al., 2021; Wegwerth et al., 2019). The cause for the switch to an exclusively freshwater life history in *S. rizeensis* remains unclear. This turns us to a question of sympatric speciation, which according to multiple recent publications is thought to be a speciation mode not less common than the “classical” pattern, wherein geographic isolation triggers early stages of speciation (Miles et al., 2018; Nunes et al., 2022).

In conclusion, our study shows that brown trout from the studied area is comprised of several distinct genetic clusters, coinciding with both separation of the sea basins and the differentiation of life histories. Fish from the Caspian Sea basin shows higher genetic differentiation among the river catchments, whereas in the Black Sea basin anadromous *S. labrax* coexists with resident form, sharing a mitochondrial haplogroup with *S. rizeensis* from northeastern Turkey. A better understanding of the genetic background and palaeoecological factors that triggered the divergence between the resident and anadromous trout in this area can be achieved with further genomic studies of the group.

## AUTHOR CONTRIBUTIONS

**Levan Ninua:** Conceptualization (equal); data curation (lead); formal analysis (equal); investigation (lead); methodology (equal); project administration (supporting); visualization (equal); writing – original draft (equal); writing – review and editing (equal). **David Tarkhnishvili:** Conceptualization (equal); formal analysis (equal); methodology (equal); supervision (supporting); visualization (equal); writing – original draft (equal); writing – review and editing (equal). **Cort Lewis Anderson:** Conceptualization (equal); methodology (equal); project administration (lead); supervision (lead); writing – review and editing (supporting).

## CONFLICT OF INTEREST STATEMENT

None declared.

## Data Availability

DNA sequences: GenBank accessions OP849668–OP849680. Microsatellite profiles: Dryad doi: 10.5061/dryad.jsxksn0fk.

## APPENDIX 1

Accession numbers of Cyt‐*b* sequences of Ponticaspian brown trout used in this study (both newly obtained and downloaded from GenBank).

**Table:**

Accession no.	Species	Sea basin	Drainage, country	Reference
FJ608987	*S. Choruhensis*	Black Sea	Coruh drainage, Turkey	Turan et al. (2009)
FJ608988	*S. Choruhensis*	Black Sea	Coruh drainage, Turkey	Turan et al. (2009)
FJ608995	*S. Choruhensis*	Black Sea	Iyidere drainage, Turkey	Turan et al. (2009)
FJ608994	*S. Choruhensis*	Black Sea	Iyidere drainage, Turkey	Turan et al. (2009)
FJ608999	*Salmo* sp.	Persian Gulf	Euphrates drainage, Turkey	Turan et al. (2009)
FJ608991	*S. riseensis*	Black Sea	Iyidere drainage, Turkey	Turan et al. (2009)
FJ608993	*S. riseensis*	Black Sea	Cayeli River, Turkey	Turan et al. (2009)
FJ608992	*S. riseensis*	Black Sea	Cayeli River, Turkey	Turan et al. (2009)
FJ608997	*Salmo* sp.	Persian Gulf	Euphrates drainage, Turkey	Turan et al. (2009)
EU492282	*Salmo* sp.	Baltic Sea	Baltic Sea, Sweden	Bautista et al. (2006)
KF985737	*S. trutta*	Black Sea	Danube drainage, Austria	Schenekar et al. (2014)
KF985738	*S. trutta*	Black Sea	Danube drainage, Austria	Schenekar et al. (2014)
FJ655773	*S. caspius*	Caspian Sea	Caspian Sea, Iran	Rezaei (2012)
EU492348	*S.trutta trutta*	Baltic Sea	Baltic Sea, Sweden	Bautista et al. (2006
FJ608989	*S. Choruhensis*	Black Sea	Coruh drainage, Turkey	Turan et al. (2009)
JN995186	*S. trutta fario*	Caspian Sea	Caspian Basin, Iran	Rezaei (2012
JX960835	*S. trutta*	Mediterranean	Göksu River, Turkey	Crete‐Lafreniere et al. (2012)
LC011387	*S. trutta caspius*	Caspian Sea	Caspian Sea, Iran	Amini and Rezaei (2017)
JX960837	*S. trutta*	Caspian Sea	Arpa River, Armenia	Crete‐Lafreniere et al. (2012)
JX960839	*S. trutta*	Adriatic Sea	Voidomatis River, Greece	Crete‐Lafreniere et al. (2012)
JX960836	*S. trutta*	Atlantic Ocean	Leksa River, Norway	Crete‐Lafreniere et al. (2012)
JX960764	*S. ohridanus*	Lake Ohrid	Lake Ohrid, Macedonia	Crete‐Lafreniere et al. (2012)
JX960841	*S. obtusirostris*	Adriatic Sea	Buna, Bosnia‐Herzegovena	Crete‐Lafreniere et al. (2012)
JX960842	*S. platycephalus*	Sea	Soğuksu Stream, Turkey	Crete‐Lafreniere et al. (2012)
JX960838	*S. marmoratus*	Adriatic Sea	Zala River, Slovenia	Crete‐Lafreniere et al. (2012)
KX594755	*S. trutta*	Unknown river	India	Singh et al. (2016)^*^
EU492108	*S. trutta*	North Sea	North Sea, the Netherlands	van Pelt‐Heerschap and Stein^*^
EU492280	*S. salar*	Baltic Sea	Baltic Sea, Sweden	Bautista et al. (2006
JX960834	*S. salar*	Atlantic Ocean	Ims, Norway	Crete‐Lafreniere et al. (2012)
KT279188	*S. trutta*	Atlantic Ocean	Dades River, Morocco	Doadrio et al. (2015)
KT279196	*S. trutta*	Atlantic Ocean	Tensift Basin, Morocco	Doadrio et al. (2015)
KT279174	*S. trutta*	Atlantic Ocean	Oum er Rbia, Morocco	Doadrio et al. (2015)
KT279171	*S. trutta*	Isli Lake	Isli Lake, Morocco	Doadrio et al. (2015)
KT279195	*S. trutta*	Mediterranean	Moulouya Basin, Morocco	Doadrio et al. (2015)
KT279191	*S. trutta*	Atlantic Ocean	Oum er Rbia, Morocco	Doadrio et al. (2015)
KT279184	*S. trutta*	Atlantic Ocean	Ziz Basin, Morocco	Doadrio et al. (2015)
KT279179	*S. trutta*	Atlantic Ocean	Oum er Rbia, Morocco	Doadrio et al. (2015)
KT279176	*S. akairos*	Ifni Lake	Ifni Lake, Morocco	Doadrio et al. (2015)
KT279177	*S. akairos*	Ifni Lake	Ifni Lake, Morocco	Doadrio et al. (2015)
KX594732	*S. trutta fario*	Unknown river	India	Singh et al. (2016)^*^
KX594730	*S. trutta fario*	Unknown river	India	Singh et al. (2016)^*^
KX594740	*S. trutta fario*	Unknown river	India	Singh et al. (2016)^*^
KX594724	*S. trutta fario*	Unknown river	India	Singh et al. (2016)^*^
NC_037399	*S. ischchan*	Lake Sevan	Caspian Basin, Armenia	Ninua et al. (2018)
MG551591	*S. ischchan*	Lake Sevan	Caspian Basin, Armenia	Ninua et al. (2018)
MG940853	*S. ischchan*	Lake Sevan	Caspian Basin, Armenia	Ninua et al. (2018)
MG029536	*S. labrax*	Black Sea	Black Sea, Georgia	Ninua et al. (2018)
MG029537	*S. labrax*	Black Sea	Black Sea, Georgia	Ninua et al. (2018)
MG029538	*S. labrax*	Black Sea	Black Sea, Georgia	Ninua et al. (2018)
MG029539	*S. labrax*	Black Sea	Black Sea, Georgia	Ninua et al. (2018)
MG029540	*S. labrax*	Black Sea	Chorokhi (Coruh), Georgia	Ninua et al. (2018)
MG029541	*S. labrax*	Black Sea	Chorokhi (Coruh), Georgia	Ninua et al. (2018)
MG029542	*S. labrax*	Black Sea	Chorokhi (Coruh), Georgia	Ninua et al. (2018)
MG029543	*S. labrax*	Black Sea	Enguri drainage, Georgia	Ninua et al. (2018)
MG029544	*S. labrax*	Black Sea	Enguri drainage, Georgia	Ninua et al. (2018)
MG029545	*S. labrax*	Black Sea	Kintrishi River, Georgia	Ninua et al. (2018)
MG029546	*S. labrax*	Black Sea	Kintrishi River, Georgia	Ninua et al. (2018)
MG029547	*S. labrax*	Black Sea	Kintrishi River, Georgia	Ninua et al. (2018)
MG029548	*S. caspius*	Caspian Sea	Kura (Mtkvari), Georgia	Ninua et al. (2018)
MG029549	*S. caspius*	Caspian Sea	Kura (Mtkvari), Georgia	Ninua et al. (2018)
MG029550	*S. labrax*	Black Sea	Rioni drainage, Georgia	Ninua et al. (2018)
MG029551	*S. labrax*	Black Sea	Rioni drainage, Georgia	Ninua et al. (2018)
MG029552	*S. ciscaucasicus*	Caspian Sea	Tergi (Terek), Georgia	Ninua et al. (2018)
MG029553	*S. ciscaucasicus*	Caspian Sea	Tergi (Terek), Georgia	Ninua et al. (2018)
OP849668	*S. labrax*	Black Sea	Chorokhi (Coruh), Georgia	This study
OP849669	*S. labrax*	Black Sea	Chakvistskali, Georgia	This study
OP849670	*S. labrax*	Black Sea	Chakvistskali, Georgia	This study
OP849671	*S. labrax*	Black Sea	Chorokhi (Coruh), Georgia	This study
OP849672	*S. labrax*	Black Sea	Enguri drainage, Georgia	This study
OP849673	*S. labrax*	Black Sea	Enguri drainage, Georgia	This study
OP849674	*S. riseensis*	Black Sea	Rioni drainage, Georgia	This study
OP849675	*S. labrax*	Black Sea	Rioni drainage, Georgia	This study
OP849676	*S. labrax*	Black Sea	Khobistskali drainage, Georgia	This study
OP849677	*S. labrax*	Black Sea	Enguri drainage, Georgia	This study
OP849678	*S. caspius*	Caspian Sea	Kura (Mtkvari), Georgia	This study
OP849679	*S. caspius*	Caspian Sea	Kura (Mtkvari), Georgia	This study
OP849680	*S. caspius*	Caspian Sea	Sulak drainage, Georgia	This study

^*^ Direct submission to NCBI.

## APPENDIX 2

Dependence of mean likelihood and delta *K*, as suggested by Evanno et al. (2005) calculated using the online application Structure Harvester (Earl & VonHoldt, 2012) for the studied samples of brown trout.

## APPENDIX 3

The allocation of the studied individuals to the clusters (*K*) inferred with STRUCTURE algorithm (considering 1 < *K* < 14). For the location numbers, see Table 1.
